# 

**Published:** 1909-03

**Authors:** 


**CONTENTS—MARCH, 1909.—VOL. XXIV, NO. 9.**

ORIGINAL ARTICLES—

Georgius Hierarchus Simmonsius—The Man in the Glass House. By G. Frank Lydston, M. D., Chicago......... 365

A Mediocrity Up-to-Date. By Henry K. Leake, A. M., M. D., Dallas, Texas.................................... 371

Women and Sanitation. By Mrs. J. M. Andrews........ 378

EDITORIAL—

Important and Notable Gathering...................... 383

EDITORIALETS.............................................. 388

CORRESPONDENCE........................................... 391

SELECTED.................................................... 392

ABSTRACTS AND SELECTIONS.............................. 394

BOOKS AND MAGAZINES....................................402

PUBLISHER'S NOTES ........................................402

